# Clinical outcomes of endovascular stenting versus bypass surgery for aortoiliac occlusive disease: a single-center retrospective cohort study

**DOI:** 10.3389/fcvm.2026.1919804

**Published:** 2026-09-21

**Authors:** Pengchao Duan, Yu-Han Qi, Jichun Zhao, Bin Huang, Ding Yuan, Tiehao Wang, Jiarong Wang

**Affiliations:** 1 Division of Vascular Surgery Department of General Surgery, West China Hospital, Sichuan University, Sichuan Province, China; 2 Department of Vascular Surgery, The First People's Hospital of Yunnan Province, The Affiliated Hospital of Kunming University of Science and Technology, Kunming, Yunnan, China

**Keywords:** aortoiliac occlusive disease (AIOD), bypass surgery, endovascular stenting, primary patency, reintervention

## Abstract

**Background:**

This study aimed to compare perioperative and follow-up outcomes between endovascular stenting and bypass surgery in patients with aortoiliac occlusive disease (AIOD).

**Materials and methods:**

This single-center retrospective cohort study included 78 patients with aortoiliac occlusive disease between January 2015 and January 2021, including 58 who underwent endovascular stenting and 20 who underwent bypass surgery. Perioperative complications, healthcare utilization, overall survival, primary patency, freedom from reintervention, and limb-related outcomes were compared between the groups.

**Results:**

Among the 78 included patients, the technical success rate was 100% in both groups. Postoperative systemic complications occurred in 3 patients (5.1%) in the ES group and 10 patients (50.0%) in the BS group (*P* < 0.001). Procedure-related complications occurred in 8 patients (13.8%) in the ES group and 1 patient (5.0%) in the BS group (*P* = 0.434). The median length of hospital stay was 7 days in the ES group and 17 days in the BS group (*P* < 0.001). No statistically significant between-group differences were observed in survival (*P* = 0.111), primary patency (*P* = 0.993), freedom from reintervention (*P* = 0.447), and freedom from any amputation (*P* = 0.750).

**Conclusion:**

In this selected retrospective cohort, endovascular stenting was associated with shorter hospitalization and fewer systemic complications than bypass surgery. No statistically significant differences in procedure-related complications, survival, primary patency, reintervention, or limb-related outcomes were observed during follow-up. These findings should be interpreted cautiously because of the small sample size, treatment-selection bias, and differences in follow-up duration.

## Highlights

- Endovascular stenting was associated with shorter hospitalization and reduced postoperative systemic morbidity.
- No statistically significant differences in survival, primary patency, reintervention, or limb-related outcomes were observed during follow-up.
- Treatment comparisons should be interpreted in the context of the retrospective design and potential anatomical selection bias.

## Introduction

Peripheral Arterial Disease (PAD) results from atherosclerosis, stenosis, or occlusion, leading to restricted blood flow in the lower extremities. Clinical presentations include claudication, rest pain, and lower limb ulcers. Over the past two decades, the incidence of PAD in China has surged by 40%, rising from 29.44 million individuals in 2000 to 41.13 million in 2020 (1–3). Aortoiliac occlusive disease (AIOD) predominantly affects individuals over 50 years old, with a higher prevalence among males. Medical intervention is crucial to prevent limb-threatening or life-threatening complications in AIOD patients.

Endovascular stenting is increasingly used as a first-line revascularization strategy for many patients with AIOD because of its lower procedural invasiveness. The Trans-Atlantic Inter-Society Consensus (TASC) recommends endovascular therapy (ET) as the preferred treatment option over open surgical repair (OSR) for TASC II A and B type AIOD lesions (4). For TASC II C and D lesions, a systematic review suggests that ET not only reduces hospitalization duration but also ensures long-term patency, with comparable outcomes for target-lesion revascularization compared to OSR (5, 6). Additionally, the latest guidelines from the European Society for Vascular Surgery emphasize the preference for endovascular stenting when performed by experienced teams (6, 7). Nevertheless, open bypass surgery remains relevant in selected patients with extensive disease, unfavorable endovascular anatomy, common femoral artery involvement, or a need for durable reconstruction.

Although endovascular-first strategies are widely used, direct real-world comparisons with open bypass remain clinically relevant. Previous studies indicate that the 24-month stent patency rate following endovascular stenting ranges from approximately 85% to 90%, while after bypass surgery, it ranges from 89% to 100% (8–10). Nevertheless, a recent study reports comparable short-term patency, survival, and limb salvage rates in complex AIOD cases (5). Additional data describing perioperative morbidity, healthcare utilization, and follow-up outcomes in selected patients may help characterize the trade-offs between these approaches.

Therefore, this study aimed to compare the perioperative characteristics, healthcare utilization, and follow-up outcomes of endovascular stenting and bypass surgery in selected patients with AIOD treated at a single center.

## Materials and methods

### Study design and setting

This single-center retrospective cohort study included patients with CTA-confirmed AIOD who underwent endovascular stenting or bypass surgery between January 2015 and January 2021 (Figure 1). The study was reported in accordance with the Strengthening the Reporting of Observational Studies in Epidemiology (STROBE) statement (11) (Supplementary Table S1). Retrospective retrieval of patient case data was conducted with approval and without the need for informed consent.

**Figure 1:**
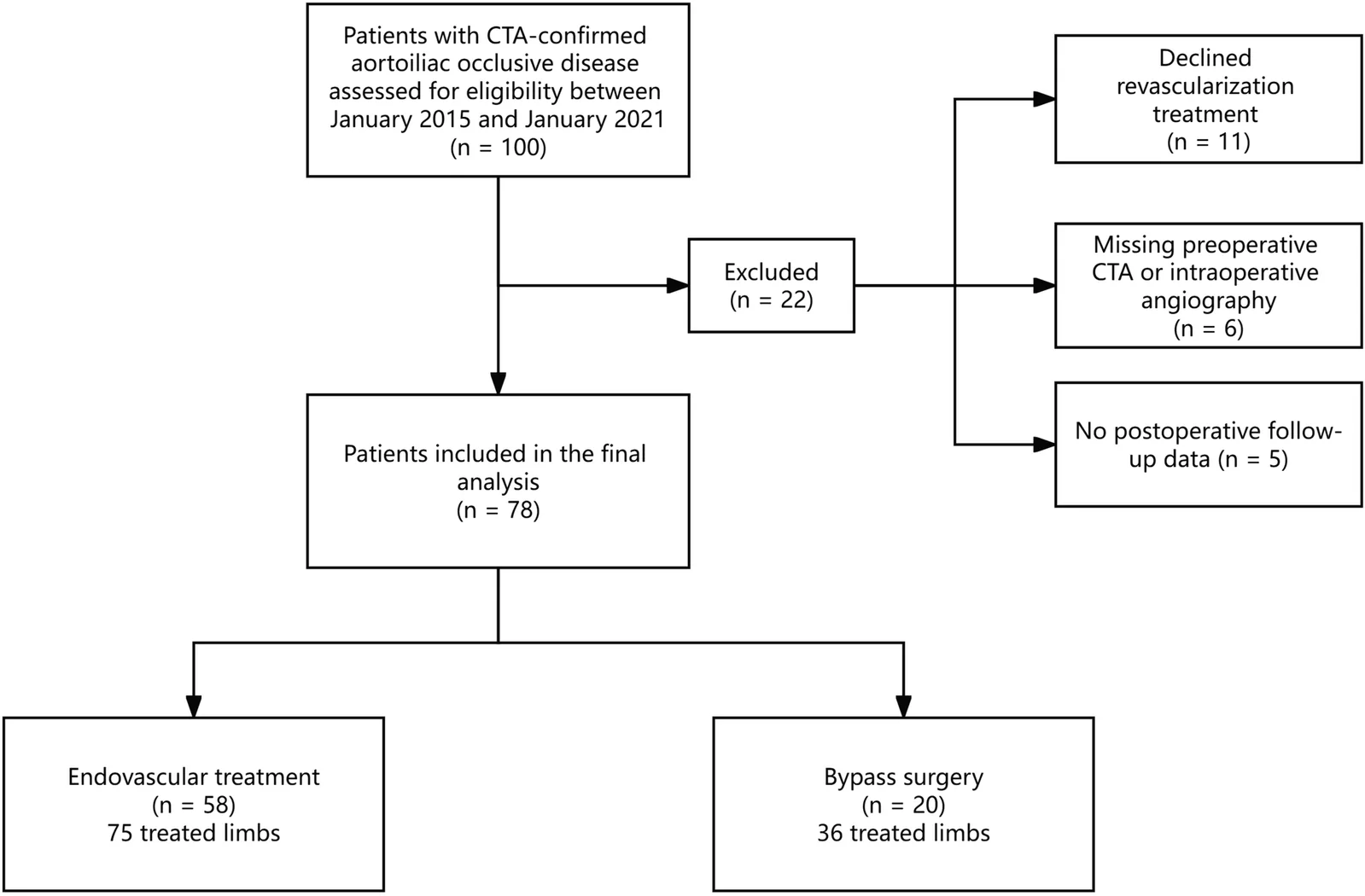
Patient selection flow diagram. Among 100 patients with aortoiliac occlusive disease assessed for eligibility between January 2015 and January 2021, 22 were excluded because they declined revascularization treatment (*n* = 11), lacked preoperative computed tomography angiography or intraoperative angiography (*n* = 6), or had no postoperative follow-up data (*n* = 5). The final analysis included 78 patients, of whom 58 underwent endovascular stenting and 20 underwent bypass surgery. The endovascular and bypass groups included 75 and 36 treated limbs, respectively.

### Participants

Patients with AIOD showing ≥70% diameter stenosis or complete occlusion on preoperative CTA were included in the study. Symptom severity was assessed using TASC II (4) and Rutherford (12) classifications. Patients meeting the following criteria were excluded: (1) Asymptomatic patients or those with mild claudication, i.e., Rutherford class 0 (asymptomatic) or 1 (mild claudication) patients. (2) History of stroke or transient ischemic attack (TIA) within the previous 3 months. (3) Inability to receive antiplatelet, anticoagulant, or thrombolytic therapy when clinically indicated. Patients with chronic atherosclerotic AIOD accompanied by an acute or subacute thrombotic component remained eligible for inclusion. Baseline information, imaging data, and other relevant data of eligible patients were obtained from the electronic medical record system. Follow-up data were collected from a designed follow-up form. Investigators assessed preoperative, intraoperative, and postoperative CTA, as well as intraoperative angiography and anatomical parameters. To ensure data reliability, 20% of the data were randomly selected for cross-checking, with measurements differing by more than 10% reassessed by a vascular surgery professor. The final cohort comprised 78 patients, including 58 who underwent endovascular stenting and 20 who underwent bypass surgery.

### Exposures

#### Treatment selection

Treatment allocation was not randomized and guided by a multidisciplinary, patient-centered decision-making process. The choice between endovascular stenting and bypass surgery was based on an assessment of the patient's clinical presentation, operative and anesthetic risk, lesion-specific anatomy, long-term prognostic implications and informed patient preferences.

#### Bypass surgery

All bypass procedures were performed under general anesthesia. Specific surgical procedures were determined based on the type and extent of arterial lesions, including aortoiliac or aortofemoral bypass (Figures 2A–D). A midline abdominal incision was made to expose the abdominal aorta, and bifurcated synthetic grafts (Gore-TEX, USA) were used for the bypass. End-to-end anastomosis was performed at the proximal end of the aorta, with end-to-side or end-to-end anastomosis at the distal end. Extra-anatomic bypass or direct iliofemoral/femorofemoral bypass was considered in cases involving unilateral iliac artery lesions.

**Figure 2:**
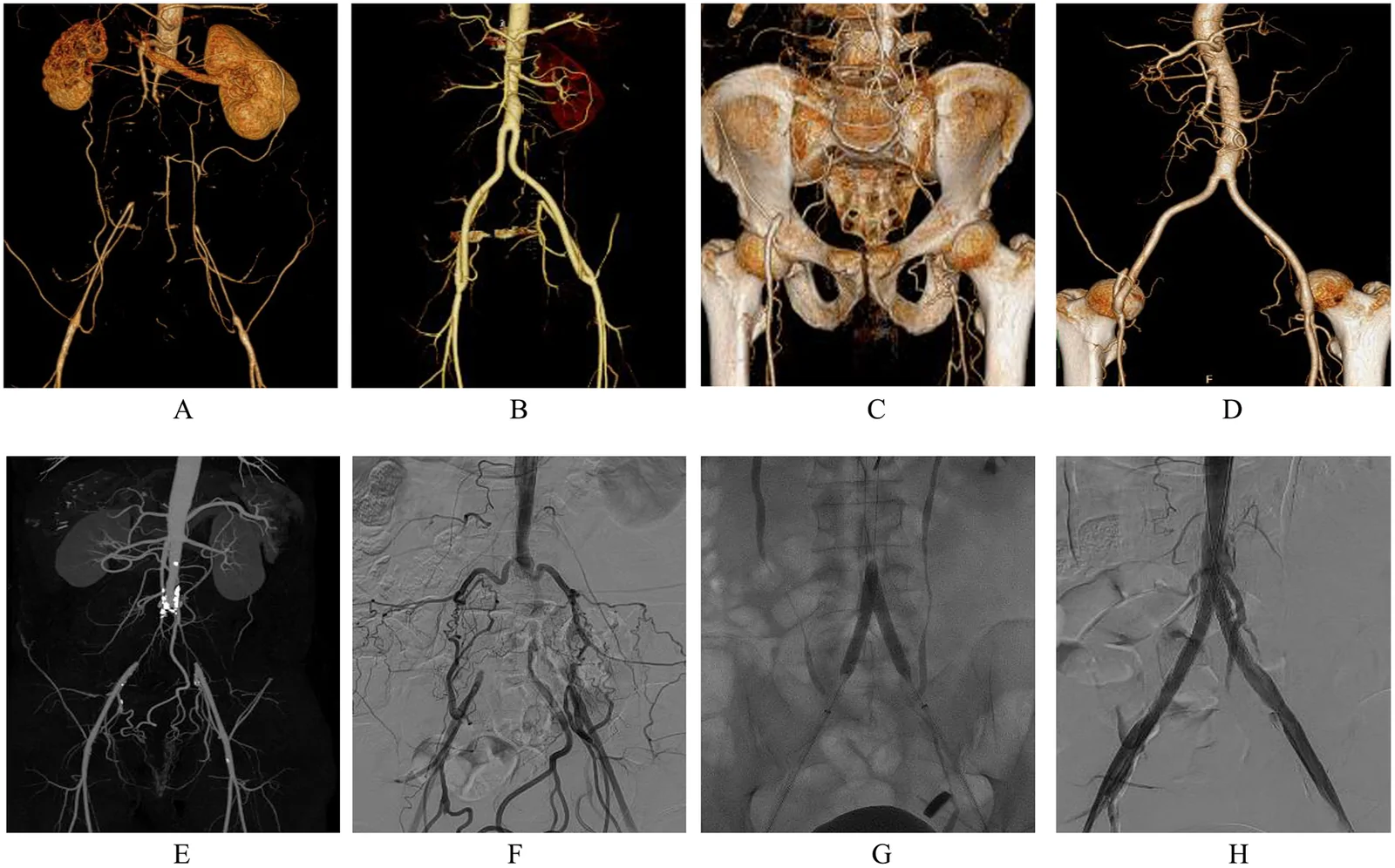
Preoperative **(A)** and postoperative **(B)** vascular reconstruction of abdominal aorta-bilateral iliac artery bypass surgery; preoperative **(C)** and postoperative **(D)** vascular reconstruction of abdominal aorta-bilateral femoral artery bypass surgery; preoperative **(E)** vascular reconstruction and intraoperative angiography **(F)** of kissing stent placement; intraoperative balloon kissing dilation **(G)**; postoperative angiography **(H)** of kissing stent placement.

#### Endovascular stenting

All patients in this group underwent preoperative CTA evaluation of the iliac artery parameters (iliac artery diameter, external iliac artery diameter, internal iliac artery diameter, lesion inflow and outflow diameter, and lesion length) to select appropriately sized abdominal aortic stents or iliac artery stents.

Preprocedural catheter-directed thrombolysis was performed in 15 patients (25.9%) with a substantial superimposed thrombotic burden. Candidates were identified by recent onset or worsening of lower-limb ischemic symptoms and CTA or angiographic evidence of intraluminal thrombus considered likely to impede lesion crossing, stent expansion, or distal perfusion. A thrombolytic catheter was positioned within the thrombosed segment, and urokinase was infused using a microinfusion pump. An initial dose of 500,000 IU was administered, with additional doses adjusted according to serial plasma fibrinogen measurements. Thrombolysis was continued for no longer than 72 h and was discontinued if the fibrinogen level decreased below 2.0 g/L. Definitive endovascular reconstruction was subsequently performed according to the residual lesion anatomy and thrombus burden.

An experienced treatment team performed endovascular stenting procedures under local anesthesia. The groin area was sterilized, and the modified Seldinger technique was used for access. Alternatively, a skin incision was made above the inguinal skin crease and below the inguinal ligament at a site with a palpable femoral artery pulsation. The femoral artery was exposed layer by layer. Intraoperative angiography was performed to reassess the target lesion. If necessary, an appropriate-sized thrombus catheter (CORDIS, USA) was used for embolic protection, and thrombus aspiration was performed to reduce the thrombus burden. For heavily calcified lesions, appropriate-sized balloons were used for pre-dilation. The stent (BARD® Fluency™, GORE® Viabahn™, or CORDIS® SMART™) was deployed to cover the entire lesion with adequate proximal and distal landing zones. Post-dilation was performed if necessary for optimal stent expansion. For complex aortoiliac lesions, kissing-stent (Figures 2E–H) or Covered Endovascular Reconstruction of the Aortic Bifurcation (CERAB) configurations were used for aortic bifurcation reconstruction.

Postoperative patients received 100 mg aspirin for antiplatelet therapy and 10 μg prostaglandin E1 injection once daily to improve microcirculation. All discharged patients received aspirin (100 mg once daily) or clopidogrel (75 mg once daily) for at least 6 months of antiplatelet therapy.

### Outcomes of interest and follow-up

The primary outcomes of this study were in-hospital systemic and procedure-related complications and primary patency. Secondary outcomes included survival, freedom from reintervention, healthcare-utilization outcomes, and technical success (bypass surgery technical success is defined as immediate graft patency with at least one vessel reaching the foot; endovascular stenting technical success is defined as successful stent deployment at the intended location with migration of <5 mm, residual stenosis <30% in the target lesion, and at least one outflow vessel reaching the foot).

Systemic complications included heart failure, respiratory failure, acute kidney injury, new-onset dialysis, infection, myocardial infarction, and in-hospital death. Procedure-related complications primarily included lower-limb thromboembolism and access-site hematoma or hemorrhage. Hematoma or hemorrhage referred to bleeding, hematoma, or pseudoaneurysm at the surgical incision or vascular access site. Primary patency was defined as uninterrupted patency of the treated arterial segment or bypass graft without clinically driven reintervention or documented occlusion during follow-up. Lower-limb thromboembolism was defined as newly detected impairment of lower-extremity perfusion or loss of arterial pulses on postoperative angiography or physical examination that had not been present preoperatively.

Patients were scheduled for follow-up at 1, 3, 6, and 12 months after discharge and annually thereafter. Follow-up was conducted primarily through outpatient visits or telephone interviews, with vascular imaging performed at least once annually. The final follow-up date was January 2026. Patients who did not attend outpatient follow-up and could not be contacted after at least two separate telephone contact attempts were considered lost to follow-up.

### Data analysis

Statistical analyses were performed using SPSS version 26.0 (IBM Corp., Armonk, NY, USA). Categorical variables are presented as numbers and percentages, whereas continuous variables are presented as mean ± standard deviation or median with interquartile range, according to their distribution. Continuous variables were compared using Student's t test or the Mann–Whitney U test, as appropriate. Categorical variables were compared using the chi-squared test or Fisher's exact test when expected cell counts were small.

Overall survival, primary patency, freedom from reintervention, and freedom from amputation were estimated using the Kaplan–Meier method. Time-to-event outcomes were calculated from the date of the index procedure to the occurrence of the corresponding event. Patients without an event were censored at the date of their last available follow-up. Cox proportional hazards regression was used to estimate hazard ratios and 95% confidence intervals. *P* < 0.05 was considered statistically significant.

## Results

In this study, a retrospective cohort of 100 patients with aortoiliac occlusive disease (AIOD) was screened, among whom 11 patients declined revascularization treatment upon admission, 6 patients lacked preoperative CT or intraoperative angiography, and 5 patients had no follow-up data. Finally, seventy-eight AIOD patients meeting the inclusion and exclusion criteria were subjected to data analysis. Among these, 20 patients, totalling 36 limbs, underwent bypass surgery, while 58 patients, with 75 limbs, underwent endovascular stenting (Figure 1). Patients in the endovascular group were older than those in the bypass group (68.8 ± 8.8 vs. 58.0 ± 12.7 years; *P* < 0.001) and had a lower preoperative eGFR [79.04 (64.68–94.60) vs. 96.48 (83.32–107.72) mL/min/1.73 m^2^; *P* = 0.007]. However, no statistically significant between-group differences were observed in preoperative or postoperative creatinine levels or postoperative eGFR. Detailed characteristics and cardiovascular risk factors are presented in Table 1. The distributions of Rutherford categories and TASC II classifications did not differ significantly between groups, although more patients in the bypass group had TASC II C or D disease (Table 1).

**Table 1:** Demographics and cardiovascular risk factors in the patients who underwent endovascular stenting (ES) and bypass surgery (BS).

Variable	ES (*n*=58)	BS (*n*=20)	*P*-value
Demographics			
Treated limbs, n	75	36	
Male sex, n (%)	53 (91.4%)	18 (90.0%)	0.852
Age, years	68.8 (8.8)	58 (12.7)	<0.001
BMI, kg/m^2^	22.51 (3.21)	22.34 (3.30)	0.882
Risk factors			
Current or former smoking, n (%)	47 (81.0%)	12 (60.0%)	0.059
Hypertension, n (%)	34 (58.6%)	8 (40.0%)	0.15
Diabetes, n (%)	14 (24.1%)	3 (15.0%)	0.393
Hyperlipidemia, n (%)	4 (6.9%)	3 (15.0%)	0.274
Cerebrovascular disease, n (%)	3 (5.3%)	1 (5.0%)	0.976
CAD, n (%)	11 (19.0%)	3 (15.0%)	0.69
CAS, n (%)	5 (10.4%)	2 (10.0%)	0.852
COPD, n (%)	8 (13.8%)	1 (5.0%)	0.289
CKD 4–5, n (%)	1 (1.7%)	1 (5.0%)	0.416
Dialysis, n (%)	1 (1.7%)	1 (5.0%)	0.416
Renal and liver function			
AST, U/L	19 (15–24.25)	17.5 (14.7–22.7)	0.783
ALT, U/L	19 (14–30)	21 (13–25.75)	0.969
PLT, ×10^9^/L	198.998 (78.2)	209.76 (74.25)	0.622
Preoperative creatinine, μmol/L	93.62 ± 31.04	111.06 ± 103.38	0.502
Preoperative eGFR, mL/min/1.73 m^2^	79.04 (64.68–94.60)	96.48 (83.32–107.72)	0.007
Clinical and anatomical classification			
Rutherford category, n (%)			0.363
2	12 (20.7%)	4 (20.0%)	
3	29 (50.0%)	7 (35.0%)	
4	11 (19.0%)	4 (20.0%)	
5	5 (8.6%)	5 (25.0%)	
6	1 (1.7%)	0	
TASC II A/B/C/D, n (%)			0.191
A class	1 (1.7%)	0	
B class	19 (32.8%)	1 (5.0%)	
C class	16 (27.6%)	8 (40.0%)	
D class	22 (37.9%)	11 (55.0%)	
C/D class	38 (65.5%)	19 (95.0%)	
Intermittent claudication, m	55 (20–300)	100 (20–200)	0.725
Aortic bifurcation/common iliac ostial involvement, n (%)	38 (65.52%)	17 (85.0%)	0.099
Follow-up duration, month	53 (15–58)	20 (10–48)	0.049

Body mass Index, CAD, coronary artery disease, CAS, coronary artery stenting, COPD, chronic obstructive pulmonary disease, CKD, chronic kidney disease, AST, aspartate aminotransferase, ALT, alanine aminotransferase, PLT, platelet, eGFR, estimated glomerular filtration rate. Categorical variables are presented as n (%). Continuous variables are presented as mean (standard deviation) or median (Q1-Q3).

All procedures were performed under local anesthesia in patients undergoing ES, while all in the BS group required general anesthesia. Among patients undergoing endovascular stenting, 25.9% received preoperative catheter-directed thrombolysis, 19.0% underwent reduction of thrombus burden, and 27.6% underwent predilation of target lesions. During the operation, 29.3% received only self-expanding covered stents (Cordis Fluency or Viabahn, USA), 62.1% received bare stents (CORDIS, SMART, USA) while 8.6% received mixed bare and covered stents (Supplementary Table S3). In the BS group, over 80% of patients underwent aorto-iliac or aorto-femoral artery bypass grafting (Supplementary Table S4).

The technical success rate was 100% in both groups. Regarding postoperative systemic complications, there were 3 cases in the ES group and 10 cases in the BS group (*P* < 0.001). Procedure-related complications occurred in 8 patients (13.8%) in the endovascular stenting group and 1 patient (5.0%) in the bypass surgery group, with no statistically significant between-group difference (*P* = 0.434). Hematoma or hemorrhage events occurred in 4 patients (6.9%) in the endovascular stenting group and 0 in the bypass surgery group, all events were associated with failure of vascular closure or compression and successfully managed with bedside measures, including manual compression, and none required surgical reintervention. Lower-limb thromboembolism occurred in 4 patients (6.9%) in the endovascular stenting group and 1 patient (5.0%) in the bypass surgery group (*P* = 1.000). Three patients in the endovascular group and the affected patient in the bypass group underwent Fogarty catheter embolectomy, whereas the remaining patient in the endovascular group was treated with thrombolysis. Limb perfusion was successfully restored in all five patients, and none had persistent ischemic symptoms before discharge. During lesion crossing or predilation, 16 patients in the endovascular stenting group developed angiographically detected, non-flow-limiting intraprocedural dissections. These events caused no distal ischemia or other clinical consequences and required no additional treatment beyond the planned procedure. They were therefore reported separately as intraprocedural technical events rather than procedure-related complications. The median length of hospital stay was 7 days in the ES group and 17 days in the BS group (*P* < 0.001). The median preoperative admission preparation days were 4.5 days for ES and 9 days for BS (*P* < 0.001). The median postoperative recovery time was 3 days for ES and 8 days for BS (*P* < 0.001). None of the patients in the ES group required postoperative ICU treatment, whereas patients in the BS group had a median ICU treatment time of 29 h (*P* < 0.001). The median treatment cost for ES was 72,812 RMB, and for BS patients, it was 93,760 RMB, with no statistical difference between the two groups (*P* = 0.102) (Table 2).

**Table 2:** Short-term outcomes and complications.

Variable	ES (n = 58)	BS (n = 20)	*P*-value
Technical Outcome			
Technical Success, n (%)	58 (100%)	20 (100%)	
Systemic Complications, n (%)	3 (5.1%)	10 (50.0%)	<0.001
MI, n (%)	0	0	
HF, n (%)	1 (1.7%)	1 (5.0%)	
Respiratory Failure, n (%)	0	1 (5.0%)	
Acute Renal Failure, n (%)	0	1 (5.0%)	
Dialysis, n (%)	0	1 (5.0%)	
Death, n (%)	0	1 (5.0%)	
Infection, n (%)	2 (3.4%）	5 (25.0%)	
Procedure-related Complications, n (%)	8 (13.8%)	1 (5.0%)	0.434
Lower limb Thromboembolism, n (%)	4 (6.9%)	1 (5.0%)	
Hematoma/Hemorrhage, n (%)	4 (6.9%)	0	
Intraprocedural technical events			
Non-flow-limiting intraprocedural dissection, n (%)	16 (27.6%)	1 (5.0%)	
Postoperative renal function			
Postoperative creatinine, μmol/L	86 (65.00–98.00)	85 (67.18–119.5)	0.793
Postoperative eGFR, mL/min/1.73m^2^	79.50 ± 18.92	83.65 ± 32.41	0.532
Healthcare utilization			
Length of Hospital Stay, days	7 (5–10)	17 (15–22)	＜0.001
Time from Admission to Surgery, days	4.5 (3–7)	9 (5–13)	＜0.001
Postoperative Hospital Recovery Time, days	3 (2–4)	8 (6–11)	＜0.001
ICU Stay, hours	0 (0–0)	29 (0–60)	＜0.001
Total hospitalization cost, RMB	72,812.28 (61,859.76–110,217.20)	93,760.88 (81,107.71–107,793.16)	0.102

MI, myocardial infarction, HF, heart failure, continuous variables are expressed as median (Q1-Q3).

During the postoperative follow-up, five patients in the ES group and four patients in the BS group died during follow-up. The survival rates at 12, 24, 36, 48, and 60 months were 98.3%, 96.3%, 91.1%, 91.1%, and 81.0% in the ES group, as well as 89.4%, 75.6%, 75.6%, 75.6%, and 75.6% in the BS group, HR, 0.36; 95% CI, 0.07–1.73. The two groups had no statistical difference (*P* = 0.111) (Figure 3A). Six patients in the ES group experienced stent stenosis or occlusion after surgery, while two patients in the BS group experienced bypass graft occlusion. The primary patency rates at 12, 24, 36, 48, and 60 months were 91.2%, 91.2%, 91.2%, 83.6%, and 83.6% in the ES group, as well as 89.7%, 89.7%, 89.7%, 89.7%, and 89.7% in the BS group, HR, 0.99; 95% CI, 0.20–4.93. The two groups had no statistical difference (*P* = 0.993) (Figure 3B). After treatment, eight ES and four BS group patients underwent reintervention for lower limb vascular stenosis/occlusion. The freedom-from-reintervention rates at 12, 24, 36, 48, and 60 months were 87.6%, 87.6%, 87.6%, 80.3%, and 80.3% in the ES group, as well as 83.7%, 76.8%, 76.8%, 76.8%, and 76.8% in the BS group, HR, 0.63; 95% CI, 0.17–2.37. The two groups had no statistical difference (*P* = 0.447) (Figure 3C). Six patients in the ES group and one patient in the BS group underwent minor limb amputation after treatment. The freedom from any amputation rates at 12, 24, 36, 48, and 60 months were 94.7%, 94.7%, 86.9%, 86.9%, and 86.9% in the ES group, as well as 100%, 92.3%, 92.3%, 92.3%, and 92.3% in the BS group, HR, 1.4; 95% CI, 0.21–9.30. The two groups had no statistical difference (*P* = 0.750) (Figure 3D).

**Figure 3:**
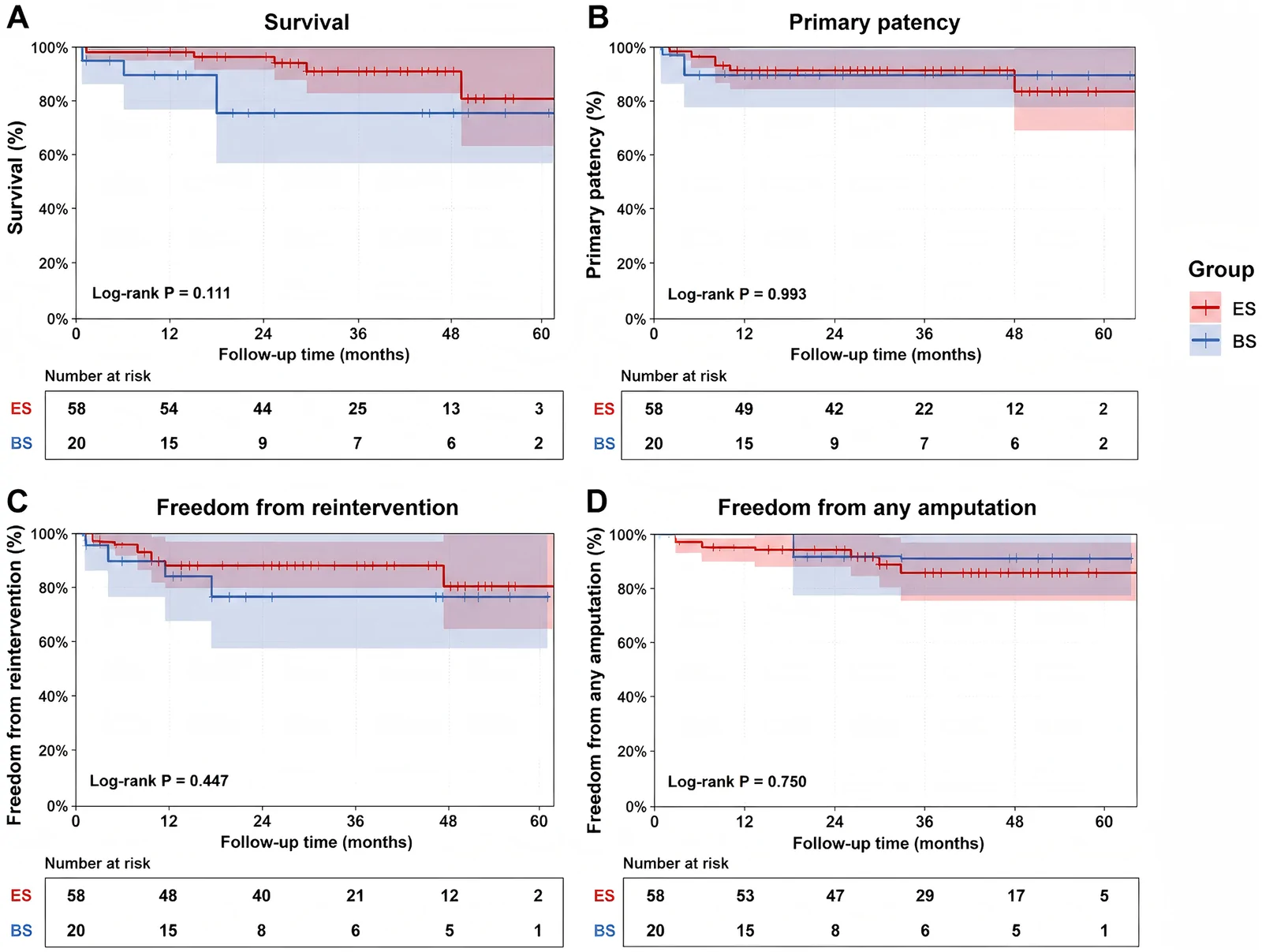
Kaplan–Meier curves for **(A)** overall survival, **(B)** primary patency, **(C)** freedom from reintervention, and **(D)** freedom from any amputation in the endovascular stenting (ES) and bypass surgery (BS) groups. Numbers at risk are shown below each panel.

## Discussion

In this retrospective single-center cohort, endovascular stenting was associated with shorter hospitalization and fewer systemic complications. No statistically significant differences in procedure-related complications, overall survival, primary patency, reintervention, or limb-related outcomes were observed during follow-up. These findings should be interpreted in light of the small sample size and non-random treatment allocation.

Previous studies and clinical practice have often considered that bypass surgery entails greater trauma (5, 13, 14). Therefore, patients selected for bypass surgery are typically younger and have fewer comorbidities related to the circulatory and respiratory systems (15, 16). In our study, the bypass surgery group appeared to have more anatomically complex disease, whereas the endovascular group was older and had lower baseline eGFR. These findings are consistent with the use of less invasive endovascular approaches in selected patients considered to be at increased operative risk. Furthermore, patients undergoing endovascular stenting had shorter hospital stays and fewer requirements for intensive care, both before and after procedure (5, 17, 18). Our study showed a 100% surgical success rate for endovascular stenting and bypass surgery. Patients undergoing bypass surgery had higher rates of systemic postoperative complications. All surgical bypass patients required surgery under general anesthesia, and some patients required further ICU care postoperatively, with a median ICU time of 29 h. In contrast, endovascular stenting patients did not require general anesthesia or postoperative ICU care, resulting in shorter preoperative preparation and postoperative in-hospital recovery times, with no statistically significant between-group difference in total hospitalization costs. These findings suggest that endovascular stenting represents a feasible and less invasive revascularization option associated with shorter hospitalization and fewer systemic complications in selected patients with AIOD. However, treatment selection should remain individualized according to anatomical suitability, operative risk, anticipated durability, and patient preference.

Compared to endovascular therapy, the potential benefit of midterm and long-term patency rates may be a significant consideration for selecting bypass surgery (19–22). In recent years, the most extensive and longest-followed study indicated that the 5-year primary patency rates of the two groups of patients were similar, with rates of 87.3% for open surgical repair and 81.4% for endovascular therapy. The 5-year limb salvage rates were 98.9% for bypass surgery and 98.4% for endovascular stenting therapy, with reintervention rates of 73.0% and 74.4%, respectively. Our study's 60-month reintervention-free rate was 80.3% for endovascular stenting and 76.8% for bypass surgery. The freedom from any amputation rate was 86.9% for endovascular stenting and 92.3% for bypass surgery at 36 months postoperatively. Our results indicate that no statistically significant differences in the evaluated follow-up outcomes were detected between groups. However, these long-term estimates should be interpreted cautiously because the number of patients remaining at risk declined substantially at later follow-up, particularly at 48 and 60 months, resulting in limited precision of the Kaplan–Meier estimates.

Compared to covered stents, bare stents may better treat AIOD by preserving blood flow in the internal iliac artery and ensuring collateral circulation. Covered stents, composed of a metal scaffold lined with polytetrafluoroethylene or polyester, can isolate plaques and intima to a certain extent, limiting the invasion of neointima and theoretically reducing the risk of restenosis (23, 24). Furthermore, a specific membrane can reduce the risk of vascular rupture during stent expansion (18). However, covered stents may lead to occlusion of side branches (such as the internal iliac artery) or thrombosis formation, requiring longer-term antiplatelet therapy. There is still controversy over the choice of stent type for AIOD patients. The latest 2020 SCAI guidelines state that bare and covered stents have high recommendation grades for patients with aortoiliac occlusion. However, further research is needed for lesions in different locations (25). In a prospective, multicenter, randomized controlled trial (COBEST trial) involving 125 patients with severe aortoiliac occlusion, patients were randomized to receive balloon-expandable covered stents or bare stents. The results showed that covered stents had similar and acceptable outcomes to bare stents in TASC II B lesions. Covered stents were superior to bare stents in short-term and long-term patency rates in TASC II C and D lesions and had lower rates of vascular reconstruction in more complex TASC II C and D lesions (26, 27). In recent years, another multicenter, randomized, single-blind controlled study compared the clinical outcomes of self-expanding covered stents and balloon-expandable bare stents in patients with aortoiliac occlusion. The freedom from restenosis rate was 84.7% in the bare stent and 89.1% in the covered stent group. The freedom from recurrence rates were 95.0% and 96.4%, respectively, while the target vessel reconstruction rates were 91.1% and 95.2%, respectively. Both stents showed no differences in effectiveness for iliac artery lesions (28). However, currently, there is limited research evaluating the efficacy and safety of balloon-expandable covered stent in the management of AIOD. Further investigation is warranted in future studies.

This study has certain limitations. Its retrospective, single-center design and non-random treatment allocation introduced substantial potential for treatment-selection bias. Propensity score matching or extensive multivariable adjustment was not considered reliable because of the small bypass cohort and limited number of outcome events. Consequently, residual measured and unmeasured confounding could not be excluded. Follow-up duration differed between groups, and the number of patients remaining at risk decreased substantially at later time points, which may have affected the comparison of long-term outcomes. Larger prospective multicenter studies with standardized treatment selection and follow-up protocols are warranted.

## Conclusion

In conclusion, endovascular stenting was associated with shorter hospitalization and fewer systemic complications than bypass surgery in selected patients with aortoiliac occlusive disease. No statistically significant differences were observed in procedure-related complications, overall survival, primary patency, freedom from reintervention, or limb-related outcomes during follow-up. Given the small and unbalanced cohort, differences in baseline characteristics and follow-up duration, and the potential for treatment-selection bias, these findings should be interpreted cautiously.

## Data Availability

The raw data supporting the conclusions of this article will be made available by the authors, without undue reservation.
